# A Clinical Practice Update for the Management of Patients With Disorders of Gut‐Brain Interaction and Altered Food Intake Behavior

**DOI:** 10.1111/nmo.70337

**Published:** 2026-05-10

**Authors:** Ayesha Shah, Nicholas J. Talley, Simon R. Knowles, Kerith Duncanson, Natasha Koloski, Jason P. Connor, Andrew S. Day, Basil Almehdawy, Chamara Basnayake, Rebecca Burgell, Sharon Carey, Charles Cock, Charlotte Daker, Vishal Kaushik, Dan Kent, Trina Kellar, Katherine Lane, Neal Martin, Allison Malcolm, Kate Elizabeth Murphy, Gemma Sharp, Caroline Tuck, Michael Jones, Nikhil Thapar, Gerald Holtmann

**Affiliations:** ^1^ Department of Gastroenterology and Hepatology Princess Alexandra Hospital Brisbane Queensland Australia; ^2^ Australian Gastrointestinal Research Alliance and NHMRC CRE in Transforming Gut Health Brisbane Australia; ^3^ Faculty of Health, Medicine and Behavioural Sciences The University of Queensland Brisbane Queensland Australia; ^4^ College of Health, Medicine and Wellbeing The University of Newcastle Newcastle New South Wales Australia; ^5^ School of Health Sciences Swinburne University of Technology Hawthorn Victoria Australia; ^6^ Alcohol and Drug Assessment Unit Princess Alexandra Hospital Brisbane Queensland Australia; ^7^ Department of Paediatrics and Child Health University of Otago Christchurch Christchurch New Zealand; ^8^ Department of Gastroenterology St Vincent's Hospital Melbourne Melbourne Victoria Australia; ^9^ Department of Medicine University of Melbourne Melbourne Victoria Australia; ^10^ Bayside Health and Monash University Melbourne Victoria Australia; ^11^ Faculty of Medicine and Health University of Sydney Sydney New South Wales Australia; ^12^ Department of Nutrition & Dietetics Royal Prince Alfred Hospital Sydney New South Wales Australia; ^13^ Dept Gastroenterology & Hepatology, Flinders Medical Centre Southern Adelaide Local Health Network & Flinders University Adelaide South Australia Australia; ^14^ Department of Gastroenterology North Shore Hospital Auckland New Zealand; ^15^ Department of Gastroenterology and Hepatology Royal Brisbane and Women's Hospital Herston Queensland Australia; ^16^ Consumer Representative Queensland Clinical Network Brisbane Queensland Australia; ^17^ Queensland Eating Disorder Service Metro North Mental Health Service Brisbane Queensland Australia; ^18^ Department of Gastroenterology Royal North Shore Hospital St Leonards New South Wales Australia; ^19^ Department of Neuroscience Monash University Melbourne Victoria Australia; ^20^ Department of Psychology Macquarie University Sydney New South Wales Australia; ^21^ Gastroenterology, Hepatology and Liver Transplant Queensland Children's Hospital Brisbane Queensland Australia; ^22^ Centre for Childhood Nutrition Research Queensland University of Technology Brisbane Queensland Australia

**Keywords:** altered food intake behavior, avoidant/restrictive food intake disorder, disorders of gut‐brain interaction, eating disorders, functional dyspepsia

## Abstract

**Background:**

Disorders of Gut‐Brain Interaction (DGBI) impact as many as one in three individuals at some stage of their lives. Clinically, patients with DGBI may present with meal‐related symptoms that progressively lead to altered food intake behaviors, resulting in subsequent weight loss and nutritional deficiencies. These patients often experience psychological distress and multi‐system co‐morbidities. This distinct subgroup can be referred to as DGBI‐induced altered food intake behavior (DGBI‐AFIB) and needs to be differentiated from the eating disorder defined in the Diagnostic and Statistical Manual of Mental Disorders, 5th edition: Avoidant/Restrictive Food Intake Behavior.

**Purpose:**

There is currently no expert consensus on the management of DGBI‐AFIB. To address this gap, a multidisciplinary, multi‐society expert panel employed a Delphi process to develop evidence‐based management guidelines for DGBI‐AFIB. The group formulated and evaluated 23 statements using the GRADE system. Experts agreed that a diagnosis of DGBI‐AFIB should be considered when gastrointestinal symptoms meet the Rome criteria for DGBI, are aggravated by food intake, and subsequently result in altered (reduced) food intake, leading to a significant (unintended) reduction in body weight. To establish a positive diagnosis of DGBI‐AFIB, relevant differential diagnoses need consideration, including organic causes/eating disorders. A multidisciplinary team approach is key to management. Treatment must align with established DGBI principles and consider each patient's specific challenges. Enteral or parenteral feeding should only be pursued in patients with life‐threatening nutritional deficiencies after all oral nutrition approaches have failed. Future research is essential to elucidate the epidemiological characteristics and risk factors for DGBI‐AFIB, thereby enhancing long‐term management.

## Key Points

- A clinical practice guideline has been developed in response to a growing number of patients with DGBI‐induced altered food‐intake behavior to guide diagnosis and management of this newly recognized condition.
- These patients often present with multisystem comorbidities, and some require enteral or parenteral nutrition.
- Managing patients with DGBI‐induced altered food‐intake behavior requires a multidisciplinary, integrated team approach (including gastroenterologists, dietitians, psychologists, and psychiatrists) to enable effective long‐term management.
- An effective treatment approach begins by establishing a positive diagnosis of DGBI‐induced altered food‐intake behavior. It rules out structural abnormalities and eating disorders as underlying causes of symptoms.
- Clinical management relies on empathetic care, includes education to create realistic patient expectations, dietitian‐supervised oral nutrition, symptom‐targeted neuromodulation, and behavioral/psychological therapies.
- Enteral or parenteral nutrition should be avoided unless there are significant nutritional deficiencies and should be used only for short periods.

## Graphical abstract

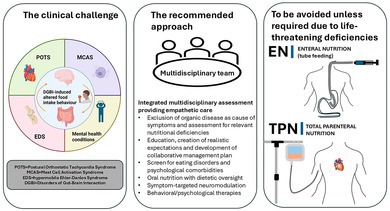

## 1 Introduction

Disorders of Gut‐Brain Interaction (DGBI) include abdominal pain‐related conditions such as irritable bowel syndrome (IBS) and functional dyspepsia (FD), which are characterized by gastrointestinal symptoms and cognitive/emotional factors without significant structural abnormalities of the digestive tract [1]. In recent years, there has been a notable increase in the number of patients with DGBI [1] and meal‐related symptoms presenting with weight loss and nutritional deficiencies due to altered (reduced) food intake behavior [2]. This group can be referred to as DGBI‐induced altered food intake behavior (DGBI‐AFIB). Their primary presentation is not an eating disorder such as anorexia nervosa, bulimia nervosa, binge eating disorder, or Avoidant/Restrictive Food Intake Disorder (ARFID). Their meal‐related gastrointestinal symptoms drive and precede the manifestation of the altered food intake behavior. These patients also often present with multi‐system comorbidities [3] and a subset request enteral feeding or parenteral nutrition [4]. Lack of awareness and definitive diagnostic criteria for this cohort results in ineffective trial‐and‐error treatments, leading to polypharmacy, increased healthcare usage, and poor quality of life [5].

### 1.1 Unmet Clinical Need

This clinical practice guideline was developed in response to the growing number of patients presenting with DGBI‐AFIB, to guide more accurate diagnosis and management of this newly recognized condition [5].

## 2 Methods

A multidisciplinary working group including gastroenterologists, dietitians, clinical psychologists, a psychiatrist, nurses, and consumer representation was convened in Australia in 2024. Using data from a rapid literature review, the working group developed draft guidelines that articulated 23 statements related to definitions, epidemiology, pathophysiology, clinical presentation and treatment. The strength of evidence for each statement was graded utilizing the Grading of Recommendations Assessment, Development and Evaluation (GRADE) system (Table 1).

**TABLE 1:** Level of evidence and grade of recommendation.

*Levels of evidence* Level I: Randomized Controlled Trials (RCTs) and Systematic Reviews of RCTs Level II: Prospective Cohort Studies Level III: Retrospective Cohort Studies and Case–Control Studies Level IV: Case Series and Poor‐Quality Cohort and Case–Control Studies Level V: Expert Opinion or Bench Research *Grades of recommendation* Grade A: High‐quality, consistent evidence from Level I studies Interventions receive a Grade A recommendation when supported by consistent, high‐quality evidence from RCTs and systematic reviews. Grade B: Moderate‐quality evidence from Level II or III studies Information/recommendation based on well‐conducted cohort or case–control studies. Grade C: Low‐quality evidence from Level IV studies Recommendations are supported by case series or poor‐quality cohort/case–control studies. Grade D: Evidence from Level V studies or inconsistent findings Recommendations essentially rely on expert opinions.

The consensus process involved a modified Delphi method [6]. The consensus team included the working group and experts from the Luminal and Pediatric Faculties of the Gastroenterological Society of Australia (GESA), the New Zealand Society of Gastroenterology (NZSG), and the Australian Society of Enteral and Parenteral Nutrition (AusPEN). The first round of online voting took place in June 2025 using an electronic online system (Microsoft Forms). The working group then analyzed the results to assess support for each statement, based on ratings of *Level of Evidence* and, where appropriate, *Grade of Recommendation*. All statements included in this guideline were required to be supported by more than 80% of the guideline group. Any ‘no votes’ or ‘non‐responses’ were counted as not supporting the respective statement. In the absence of high‐level clinical evidence, the modified Delphi process is used to achieve expert consensus on a pragmatic, clinically usable definition for guideline implementation across diverse settings. Moreover, we recognize that much of the underpinning knowledge cited in the manuscript arises from the broader DGBI literature, rather than studies of the newly proposed operational definition as these are not currently available. The resulting DGBI‐AFIB guideline was endorsed by the GESA, NZSG and AusPEN. This current document summarizes the key findings and recommendations summarized in this manuscript.

The DGBI‐AFIB guideline is intended to support both adult and pediatric healthcare professionals, particularly multidisciplinary teams working with gastroenterologists, in providing appropriate and consistent care for patients with DGBI‐AFIB. This document should be evaluated alongside existing national [7, 8, 9, 10, 11, 12, 13] and international consensus documents [1, 14] on DGBI, as well as guidance on managing eating disorders [15, 16, 17], particularly ARFID.

### 2.1 Clinical Presentation of Patients With DGBI and Altered Food Intake Behavior

Statement 1 In population‐based cohorts, symptoms of dyspepsia (either with nausea and vomiting or meal‐related symptoms) can be associated with significant unintentional weight loss (e.g., > 5% of body weight within 12 months).

Level of evidence: II; Grade of Recommendation: B; Accepted by 92.3%.

Population‐based cohorts [18] reveal that subjects with DGBI (and symptoms such as nausea, vomiting, and meal‐related symptoms with and without psychological distress) often experience significant (≥ 5%) weight loss [18, 19].

Statement 2 Weight loss (> 5% of body weight) due to altered food intake behavior resulting from meal‐related symptoms can occur in patients with DGBI, such as functional dyspepsia with postprandial distress syndrome.

Level of evidence: II; Grade of Recommendation: B; Accepted by 92.3%.

Patients with DGBI often experience a range of eating‐ or meal‐related symptoms that significantly impact their eating behaviors and overall well‐being [20]. FD includes two overlapping subtypes: pure Postprandial Distress Syndrome (PDS) and the PDS‐Epigastric Pain Syndrome (EPS), which together account for 82% of all FD cases.

In a study involving adolescents with IBS [21], 92% reported symptoms associated with eating, with 95% attributing them to specific foods. Some avoided these trigger foods altogether, even skipping meals despite hunger. Thus, in a subgroup of patients, severe symptoms prompted changes in their eating habits, modifying and reducing their food intake, and ultimately resulting in weight loss and experiencing other effects such as social isolation and negative impacts on quality of life. A recent systematic review [22] highlights that the prevalence of disordered eating in gastrointestinal disorders is 33.2% (95% CI: 0.25–0.41), with 30.2% (95% CI: 0.21–0.40) specifically in IBS.

### 2.2 Diagnostic Approaches for Patients With DGBI and Altered Food Intake Behavior

#### 2.2.1 Diagnosis of DGBI and Altered Food Intake Behavior

Statement 3 ‘DGBI and altered food intake behaviour’ are characterized by gastrointestinal symptoms that fulfill all the below:

- Meet the current Rome criteria for DGBI (e.g., functional dyspepsia or irritable bowel syndrome)
- Are aggravated by food intake and result in altered (reduced) food intake
- Result in subsequent (unintended) reduction of body weight (> 5% of body weight over 6 to 12 months)
- Predate the altered food intake behavior and weight loss
- Have been deemed not to represent an eating disorder, which has been ruled out or does not explain the clinical presentation.

Level of evidence: V; Grade of Recommendation: D; Accepted by 85.5%.

Defined criteria are essential to differentiate patients with DGBI‐AFIB from other gastrointestinal disorders that can cause disordered eating, with or without weight loss. The Rome IV criteria define DGBI as disorders characterized by chronic or recurrent gastrointestinal symptoms resulting from alterations in gastrointestinal function and/or the interaction between the gastrointestinal system and the central nervous system [1]. Currently altered (reduced) food intake behavior in patients with DGBI can be a direct consequence of a severe DGBI manifestation, frequently against the background of other comorbidities (e.g., anxiety/depression) [23, 24]. Reduced food intake, in the context of altered food intake behavior, refers to a sustained decrease in the amount or frequency of food consumed, beyond occasional skipped meals. In DGBI‐AFIB, meal‐related symptoms precede the manifestation of weight loss, which helps differentiation from reduced food intake in patients with eating disorders that can subsequently result in meal‐related symptoms and alterations of gastric emptying [25]. To assist recognition and differentiation of patients with ‘DGBI and altered food intake behaviour’ in the routine clinical setting, it is recommended to apply the established Rome Criteria for DGBI [1, 26] with subtype (e.g., criteria for FD with PDS [27]) and its sequence in relation to any clinically significant weight loss operationalized as an arbitrary but consensus‐based weight decline of ≥ 5% over the preceding 6–12 months. ‘Weight loss’ was chosen instead of ‘nutritional deficiencies’ because it is a practical and uniform criterion for the identification of these patients across diverse clinical settings. Nutritional deficiencies often accompany weight loss are also clinically important but were not adopted as a primary criterion because they are heterogeneous in presentation, influenced by prior diet and comorbidities, with inconsistent availability and variability in laboratory testing, (Statement 3 and Table 2).

**TABLE 2:** Clinical features and criteria allowing differentiation of disorders of gut‐brain interaction and altered food intake behavior (DGBI‐AFIB) vs. key eating disorders (Diagnostic and Statistical Manual of Mental Disorders, 5th Edition (DSM‐5‐TR)).

	DGBI‐AFIB	DSM‐5‐TR avoidant/restrictive food intake disorder (ARFID)	DSM‐5‐TR anorexia nervosa (AN)	DSM‐5‐TR bulimia nervosa (BN)	DSM‐5‐TR binge eating disorders (BED)	DSM‐5‐TR, pica
Patients meet the criteria for DGBI (Rome IV criteria), predating the onset of altered food intake behavior.	Required	Does not exclude ARFID	Does not exclude AN	Does not exclude BN	Does not exclude BED	Not a typical feature of Pica
The DGBI‐related symptoms are severe or very severe, defined by their impact on daily life. Severe symptoms cannot be ignored by the patient and impact daily life.	Required	Does not exclude ARFID	Does not exclude AN	Does not exclude BN	Does not exclude BED	Not a typical feature of Pica
Gastrointestinal symptoms are triggered or aggravated by food intake and are consistent with a DGBI before the onset of the altered food intake. These symptoms are the primary (leading) cause for the restricted food intake.	Required	Does not exclude ARFID	May exclude AN	May exclude Excludes BN	Excludes BED	Not a typical feature of Pica
Eating or feeding disturbance (e.g., apparent lack of interest in eating; avoidance based on the sensory characteristics of food; concern about aversive consequences of eating), failure to meet nutritional and/or energy requirements associated with one or more of the following: weight loss, inability to achieve expected weight gain, dependence on enteral feeding or nutritional support, interference with psychosocial functioning.	May co‐occur but may not fully explain the eating or feeding disturbance.	Required	Not a typical feature of AN	Not a typical feature of BN	Not a typical feature of BED	Not a typical feature of Pica
Restriction of energy intake leads to significantly low body weight, an intense fear of gaining weight, and a distorted body image (restrictive vs. binge eating purge behavior).	Not a typical feature but restrictions of caloric intake can occur.	Not a typical feature of ARFID	Required	Not a typical feature of BN	Not a typical feature of BED	Not a typical feature of Pica
Recurrent episodes of binge eating, with consumption of excessive amounts of food with a sense of lack of control, followed by inappropriate compensatory behaviors (such as self‐induced vomiting, excessive exercise, or misuse of laxatives).	Not a typical feature	Not a typical feature of ARFID	Not a typical feature of AN	Required	Not a typical feature of BED	Not a typical feature of Pica
Recurrent episodes of binge eating, defined as consuming an excessive amount of food in a discrete period, accompanied by a sense of loss of control overeating.	Not a typical feature	Not a typical feature of ARFID	Not a typical feature of AN	Not a typical feature of BN	Required	Not a typical feature of Pica
Persistent consumption of non‐nutritive, non‐food substances.	Not a typical feature	Not a typical feature of ARFID	Not a typical feature of AN	Not a typical feature of BN	Not a typical feature of BED	Required
Repeated regurgitation of food, which may be re‐chewed, re‐swallowed, or spit out. It is considered a learned behavior that is classified as a DGBI.	Not a typical feature	Not a typical feature of ARFID	Not a typical feature of AN	Not a typical feature of BN	Not a typical feature of BED	Not a typical feature of PICA

Following a GI infection, a patient may develop meal‐related symptoms consistent with post‐infectious functional dyspepsia (FD). This may lead to temporary changes in eating behavior that are often resolved spontaneously, with no meaningful weight loss or nutritional consequences. In contrast, patients with DGBI‐AFIB exhibit a persistent, food‐avoidant pattern where intake is significantly reduced and aggravated by meals, leading to unintended weight loss over time. This trajectory can culminate in the need for enteral or parenteral feeding to maintain nutrition. Recognizing this distinction is important for prognosis and guiding multidisciplinary management, including GI–psychology and nutritional interventions.

#### 2.2.2 Delineation of DGBI and Altered Food Intake Behavior and Eating Disorders

Statement 4 Eating disorders need to be assessed and considered in the differential diagnosis of DGBI‐AFIB.

Level of evidence: II; Grade of Recommendation: B; Accepted by 92.3%.

In the context of patients presenting with altered food intake behavior, it's essential that DGBI‐AFIB and DSM‐5‐TR defined eating disorder (Table 2) (e.g., anorexia nervosa, bulimia nervosa or ARFID) are considered as differential diagnoses. In DGBI‐AFIB, chronic gastrointestinal symptoms, exacerbated by food intake and meeting DGBI criteria occur before noticeable weight loss due to food avoidance. Therefore, to distinguish DGBI‐AFIB from an eating disorder, it's crucial to consider the patient's history and the progression of symptoms.

Importantly, DGBI‐AFIB and an eating disorder (ARFID) can co‐exist in a subset of patients, and the temporal relationship between gastrointestinal symptoms and abnormal eating behavior can be difficult to determine retrospectively.

It may be possible to distinguish DGBI‐AFIB from avoidant/restrictive food intake disorder (ARFID) by anchoring the diagnosis of ARFID clearly within established DSM‐5‐TR criteria, while recognizing that symptom‐driven dietary restriction in DGBI may overlap but does not constitute an eating disorder. ARFID requires a persistent disturbance in eating leading to clinically significant consequences—such as weight loss (or faltering growth), nutritional deficiency, dependence on enteral feeding or oral nutritional supplements, and/or marked psychosocial interference—occurring in the absence of body image disturbance and not better explained by another medical condition alone. In contrast, individuals with DGBI‐AFIB demonstrate restricted or avoidant intake that is closely linked to gastrointestinal symptoms (e.g., pain, bloating, nausea), typically remaining circumscribed to symptom‐specific food avoidance rather than a broader disturbance in feeding behavior. While these conditions can overlap and exist along a spectrum, it is important not to rely on psychosocial impairment as the sole point of differentiation. Some individuals with ARFID may meet biological criteria (e.g., weight loss or nutritional compromise) with limited overt psychosocial impairment, whereas in DGBI the degree of restriction is more proportionate to symptom management and does not reflect the pervasive feeding disturbance characteristic of ARFID.

Indeed, more than 94% of patients with untreated Anorexia Nervosa meet criteria for FD, and over 50% also meet criteria for IBS [28]. Increased prevalence of symptoms consistent with DGBI is observed in other eating disorders [29]. The severity of gastrointestinal symptoms and number of DGBI diagnoses are influenced by eating disorder symptom severity, with those reporting consistently severe symptoms meeting criteria for more than three DGBI, while those with less severe symptoms meet criteria for one [29]. Furthermore, malnutrition and restrictive food intake behaviors can lead to meal‐related symptoms and gastrointestinal physiology changes, such as delayed gastric emptying [30], similar to those seen in patients with gastroparesis. Most patients with eating disorders experience some improvement in their gastrointestinal symptoms with weight restoration [31, 32], however, DGBI diagnosis may change throughout the course of eating disorder treatment and gastrointestinal symptoms can persist beyond eating disorder recovery for some [33]. It remains unclear to what extent nutritional rehabilitation, reduction in eating disorder symptoms, or improvement in pathophysiological processes in DGBI (regardless of eating disorder status) contribute to improved gastrointestinal symptoms within this group [34].

Complicating the matter, standardized eating disorder screening questionnaire tools are likely to be invalid in patients with concurrent gastrointestinal conditions, overestimating the prevalence of disordered eating [22]. For example, in a cohort study utilizing a standardized screening tool (Nine‐Item ARFID Screen) for ARFID involving 289 adults with well‐established organic gastrointestinal conditions, including achalasia, celiac disease, eosinophilic esophagitis, and inflammatory bowel disease, 53.7% met current diagnostic criteria for ARFID [35], highlighting the current limitations of such tools [35].

DGBI‐induced AFIB is not intended to replace ARFID or other eating disorders but rather serves to describe patients that have severe alteration in feeding behavior, as a direct result of food induced GI symptoms, which leads to adverse medical consequences but who do not neatly fit into DSM‐V eating disorder/ARFID criteria (i.e., nil body dysmorphia, desire to gain weight, sensory aversion). Indeed, some individuals with DGBI have meal‐related symptoms that lead to reduced food intake or avoidance of certain foods and whilst significant does not evolve into the broader behavioral and psychological features which are characteristic of ARFID and this cohort may be described as DGBI‐AFIB.

The differentiation of DGBI‐AFIB and DSM‐5‐TR defined eating disorders, including ARFID, is important considering the specific evidence‐based treatment options that may be available for patients with eating disorders separate to approaches that are appropriate for patients with DGBI‐AFIB. Ultimately, the treatment modalities available for the patient are more important than the diagnostic label. For example, some sufferers may find it more acceptable to engage with a diagnosis of DGBI‐AFIB rather than an eating disorder diagnosis. Characteristics of patients who meet the criteria for DGBI‐AFIB and those with other specific eating disorders are summarized in Table 2.

#### 2.2.3 Comorbidities in Patients With DGBI and Altered Food Intake Behavior

Statement 5 Anxiety, depression, hypermobility spectrum disorders (HSDs), postural orthostatic tachycardia syndrome (POTS), mast cell activation syndrome (MCAS) and vascular compression syndromes are comorbidities that appear to occur in patients with DGBI‐AFIB.

Level of evidence: V; Grade of Recommendation: D; Accepted by 88.5%.

Statement 6 The diagnosis of HSD, POTS, MCAS or vascular compression syndromes in patients with DGBI‐AFIB should always meet contemporary, evidence‐based criteria, and these conditions should not be diagnosed based solely on clinical suspicion or self‐diagnosis.

Level of evidence: V; Grade of Recommendation: D; Accepted by 88.5%.

Anxiety and depression are present in more than 50% of patients with DGBI [23, 36, 37, 38]. A systematic review found that anxiety and depression, along with female sex, younger age, gastrointestinal symptom severity, and lower quality of life, were associated with disordered eating [39].

Patients with DGBI‐AFIB often present with a cluster of systemic (e.g., rheumatologic, immunologic, and cardiovascular) comorbidities. Notable among these are hypermobility spectrum disorders (HSD) [40], postural orthostatic tachycardia syndrome (POTS) [41], mast cell activation syndrome (MCAS) [42, 43] and vascular compression syndromes, for example, median arcuate ligament syndrome (MALS) [44], each of which are considered rare in the general population with prevalence rates < 1% for HSD [45]; < 2% for MCAS; < 1% for POTS [46] and < 0.01% for MALS [47].

Moreover, a growing body of literature highlights the co‐occurrence of MCAS, POTS, and HSD [3]. Diagnosing these conditions presents significant challenges, largely because the underlying pathophysiology of each remains incompletely understood and the diagnostic criteria are continually evolving [3] (Statements 5 and 6). However, a detailed discussion of these rare comorbidities, often linked with DGBI‐AFIB and frequently based on self‐diagnosis and clinical suspicion, falls outside the scope of this article.

#### 2.2.4 Clinical Assessment of Patients With Altered Food Intake Behavior

Statement 7 Clinical outcomes and patient characteristics in those with suspected DGBI‐AFIB should be documented where possible, using validated instruments (e.g., patient reported outcome measures).

Level of evidence: II; Grade of Recommendation: B; Accepted by 85.5%.

The initial evaluation of patients with suspected DGBI‐AFIB requires a comprehensive history covering the onset, duration, and nature of changes in food intake, along with gastrointestinal or psychological symptoms and relevant comorbidities.

A physical examination should assess for signs of malnutrition, dehydration, and any organic gastrointestinal diseases. Essential anthropometric measurements (weight, height, Body Mass Index (BMI), and body composition) must be obtained to assess nutritional status, with emphasis on documenting weight loss or failure to gain weight.

Patients with DGBI‐AFIB due to meal‐related gastrointestinal symptoms should undergo appropriate investigations to rule out organic gastrointestinal causes for their symptoms [48]. For example, given the link between food intake and the manifestation of symptoms, it is crucial to consider the diagnosis of food allergies [49]. Again, self‐perception of food allergies rather than a confirmed diagnosis is common, especially in non‐IgE mediated food allergies given a lack of tests to definitively diagnose them. In children, recommended practice is for an appropriate short‐term diagnostic elimination diet to observe for symptom resolution, followed by food reintroduction to assess for symptom recurrence [50, 51].

A psychological assessment is recommended for patients with suspected psychological comorbid conditions including anxiety, depression, obsessive‐compulsive disorder and specific phobias (e.g., specific phobias associated with vomiting like emetophobia). These conditions can significantly impact quality of life and adversely influence food intake due to an exaggerated fear of experiencing pain and discomfort after a meal. Validated instruments to assess gastrointestinal symptoms and screening for depression, anxiety, somatic complaints, gastrointestinal‐specific visceral anxiety, and pain catastrophizing are beneficial [38, 52, 53, 54] to evaluate the patient's condition and monitor clinical progress. The DSM‐5‐TR specifies that in ARFID the eating disturbance does not result from a medical condition or mental health disorder (or is disproportionate/exceeds what would be normally expected) affecting their food intake behavior. However, it's important to recognize that eating disorders can co‐occur with DGBI.

In the routine clinical setting, screening for gastrointestinal and extraintestinal symptoms using validated instruments ensures: (a) consistent data collection across patients; (b) coverage of a wide range of symptoms; (c) patients can effectively articulate severity of specific symptoms; (d) establishment of baseline measurements to evaluate treatment effectiveness during follow‐up visits; and (e) aggregated data that aids research and informs quality improvement measures within clinical practice (Statement 7).

In many cases, it is not clinically possible to determine whether the gastrointestinal symptoms preceded the altered food intake behavior or emerged during the disordered eating behaviors. Regardless, the association between the gastrointestinal symptoms and the act of eating, often reinforces food avoidance.

In settings where feasible, a thorough dietetic assessment is recommended for patients with suspected DGBI‐AFIB. This dietitian‐initiated assessment would include evaluating the individual's food‐related knowledge and skills, and social environment that may influence dietary intake and capacity to follow dietary recommendations. A detailed dietary intake assessment (e.g., 7‐day food diary, 24 h recall or food frequency questionnaire such as the Comprehensive Nutrition Assessment Questionnaire) [55] with comparison to estimated nutrition requirements provides a basis for identifying nutritional inadequacies and guiding targeted dietetic intervention [56].

#### 2.2.5 Diagnostic Measures to Identify Altered Gut Function

Statement 8 The results of gut function testing need to be interpreted with caution in the context of concomitant medication (anticholinergic drugs, opioids, proton pump inhibitors), nutritional status (e.g., malnourishment or starvation) and the underlying symptoms before another clinical diagnosis (e.g., gastroparesis, small intestinal bacterial overgrowth) is established in these patients.

Level of evidence: II; Grade of Recommendation: C; Accepted by 84.6%.

The diagnosis of DGBI‐AFIB should be made clinically. While function testing can provide insights into underlying disease mechanisms, it is not necessary for establishing the diagnosis (Statement 8). Further, some changes of gastrointestinal function can result from altered food intake rather than being causative. Thus, the role of function testing for the routine assessment and management of patients with DGBI, in the context of DGBI‐AFIB, remains to be determined.

### 2.3 Management Strategies for Patients With DGBI and Altered Food Intake Behavior

#### 2.3.1 Importance of an Integrated Multidisciplinary Team (iMDT) Approach

Statement 9 The aims of treatment for patients with DGBI‐AFIB are to:

– Reduce symptoms (e.g., meal‐related pain, vomiting, distension, constipation/diarrhea, bloating/distension)
– Reduce overall morbidity and mortality
– Facilitate nutritional repletion and achievement of a body weight that supports metabolic and functional recovery.
– Reduce psychological distress (e.g., anxiety and depression) and improve quality of life
– Avoid treatment‐related complications.

Level of Evidence: V; Grade of Recommendation: D; Accepted by 92.3%.

Statement 10 In the absence of data from clinical trials specifically targeting patients with DGBI‐AFIB, treatment and management for these patients should follow the established principles for patients with DGBI. Besides general measures, these treatments include pharmacologic (e.g., neuromodulators) and non‐pharmacologic (dietary, psychological and behavioral) interventions, preferably with a multidisciplinary team.

Level of Evidence: V; Grade of Recommendation: D; Accepted by 92.3%.

Statement 11 Patients with DGBI‐AFIB should be managed by providers with the required specialized knowledge (e.g., gastroenterologists, psychiatrists, psychologists and dietitians with special expertise on the management of patients with DGBI and/or gastrointestinal disorders).

Level of Evidence: V; Grade of Recommendation: D; Accepted by 88.5%.

While many patients with DGBI receive appropriate treatment in primary care, patients with severe DGBI manifestations require management that involves specific expertise, ideally delivered by an iMDT. Because altered gut‐brain interactions and impaired gut function play a crucial role in symptom manifestation and potential medical complications, the specialized knowledge of gastroenterologists (both adult and pediatric), allied health professionals (dietitians, psychologists), and psychiatrists is essential for the care of these patients. An MDT approach is seen to provide superior outcomes in patients with DGBI [36, 57, 58] that are likely helpful for DGBI‐AFIB. Appropriate models of care, especially for patients in rural and remote regions, remain a challenge. Therefore, it is often most suitable for management to be coordinated by a primary care physician who is guided by an expert team in a hub‐and‐spoke model. Effective medication management and medical monitoring are the responsibilities of the gastroenterology team, in close collaboration with primary care physicians (Statements 10 and 11).

### 2.4 Dietary Interventions, Enteral Feeding and Parenteral Nutrition

Statement 12 For patients with DGBI‐AFIB, enteral and/or parenteral nutrition should be avoided.

Level of evidence: V; Grade of Recommendation: D; Accepted by 92.3%.

Statement 13 For all malnourished patients with DGBI‐AFIB, oral nutrition supplements should be comprehensively trialed with dietetic oversight before consideration of enteral or parenteral nutrition.

Level of evidence: V; Grade of Recommendation: D; Accepted by 92.3%.

Statement 14 If enteral nutrition needs to be considered to address malnutrition in patients with DGBI‐AFIB, it is a short‐ to medium‐term (days or up to 4 weeks) measure aimed at stabilizing nutritional status, preventing further decline, and supporting a transition to adequate oral intake as soon as possible.

Level of evidence: V; Grade of Recommendation: D; Accepted by 92.3%.

Statement 15 Surgically implanted feeding tubes should be avoided in the management of patients with DGBI‐AFIB, since enteral feeding should not be routinely used and, if applied, should only be temporary and used for the shortest possible period.

Level of evidence: V; Grade of Recommendation: D; Accepted by 92.3%.

Statement 16 Only if oral feeding and enteral nutrition prove to be unsuccessful and there is severe, life‐threatening malnutrition, short‐term, peripheral intravenous fluids may be administered in patients with DGBI‐AFIB.

Level of evidence: V; Grade of Recommendation: D; Accepted by 92.3%.

Statement 17 When enteral or parenteral nutrition is considered in patients with DGBI‐AFIB to address life‐threatening nutritional deficiencies, a second opinion from an expert, preferably with experience in the management of patients with complex DGBI or intestinal failure, is recommended to support adherence to clinical standards, manage patient expectations effectively, and minimize the risk of harm.

Level of evidence: V; Grade of Recommendation: D; Accepted by 92.3%.

Statement 18 If enteral or parenteral nutrition is considered in patients with DGBI‐AFIB to address life‐threatening nutritional deficiencies, the patients should be managed at centres with appropriate support from a multidisciplinary team with medical (gastroenterology, psychiatry), allied health (dietetics, psychology) and nursing staff with expertise in both enteral and parenteral nutrition and the management of patients with complex DGBI.

Level of evidence: V; Grade of Recommendation: D; Accepted by 92.3%.

Statement 19 If enteral or parenteral nutrition is required to address life‐threatening nutritional deficiencies in DGBI‐AFIB, oral feeding should be continued and enteral or parenteral nutrition discontinued as soon as oral feeding is sufficient to meet nutritional requirements.

Level of evidence: V; Grade of Recommendation: D; Accepted by 92.3%.

Dietary interventions are key in managing DGBI symptoms. They are based on a detailed dietetic assessment including the identification of dietary triggers of symptoms and use this to implement appropriate dietary interventions. In patients with DGBI‐AFIB, non‐restrictive dietary interventions to target symptom reduction such as the National Institute of Clinical Excellence (NICE) diet or Mediterranean diet are more appropriate than restrictive dietary interventions [56]. Restrictive diets are contra‐indicated in patients with DGBI‐AFIB and concurrent/secondary malnutrition. While a low Fermentable Oligosaccharides, Disaccharides and Polyols (FODMAP) diet is commonly used in IBS, a modified “FODMAP‐gentle” approach [59] might be more appropriate in DGBI‐AFIB to minimize risk of reinforcing harmful restrictive eating [60, 61]. Patients who have self‐implemented diets may be at an increased risk of DGBI‐AFIB as they; might avoid whole food groups, or undergo Diet Stacking [62] whereby they follow multiple simultaneous restrictive diets, with a subsequently more significant impact on nutritional health [63].

Dietitians play an important role in patients with DGBI‐AFIB to rebuild food range and diet diversity, using structured approaches such as personalized education, nutrition counseling, gradual food reintroduction, meal planning with a variety of ingredients, and incorporating food exposure techniques. Optimizing timing of dietary intake, as per the NICE guidelines, and avoiding meal skipping can be an important dietary intervention strategy to optimize gut function. In patients with DGBI‐AFIB, oral nutrition support in the form of high energy/protein oral nutrition supplements, or high energy/protein meals and snacks may be implemented in a personalized manner to meet nutritional needs.

#### 2.4.1 Enteral Nutrition

In malnourished patients with DGBI and a functioning gastrointestinal tract but unable to achieve adequate oral intake due to gastrointestinal symptoms, initiation of enteral tube feeding can be tempting. The European Society for Clinical Nutrition and Metabolism (ESPEN) Clinical Guidelines for Home Enteral Nutrition define inadequate nutritional state as a patient's inability to eat for a week or energy intake < 60% of estimated requirements for 1–2 weeks (usually < 10 kcal/kg/d or a lack of 600–800 kcal/day) [64]. However, caution is advised when commencing enteral feeding in patients with DGBI without malnutrition or objective biochemical abnormalities, as it can pose significant risks of iatrogenesis, including exacerbation of symptoms, psychological dependence on enteral feeding and complications related to tube‐feeding [65]. There is also a lack of evidence for improved global function, quality of life or symptoms with enteral feeding in this population [66, 67, 68].

Thus, enteral feeding in patients with DGBI‐AFIB is only justifiable under exceptional circumstances in malnourished patients when other measures have been exhausted and appropriate treatment has failed to address a life‐threatening situation. If enteral nutrition is considered in these patients, it is a short‐ to medium‐term (days or up to 4–6 weeks) measure aimed at stabilizing nutritional status, preventing further decline, and supporting a transition to adequate oral intake [69]. Invasive interventions such as feeding via a percutaneous gastrostomy or jejunostomy should always be preceded by more conservative approaches to avoid escalation to interventions with a greater risk of iatrogenesis [70] until the effects of long‐term treatment are understood. Examples of appropriate interventions include specialist dietetic counseling and oral dietary adjustments, which may include oral nutrition supplements to maximize *effortful oral feeding*, or, under exceptional circumstances, time‐limited naso‐enteral tube trials of 4–6 weeks with maximal multidisciplinary input to optimize effective weaning to an oral diet [64, 65, 66, 69].

While adult‐specific weaning protocols are under‐researched, the pediatric literature highlights the importance of an MDT approach [65]. These principles may be adapted for adults into a structured, MDT approach to gradual oral diet reintroduction, prioritizing sustained oral intake and addressing barriers such as food intolerances, appetite, feeding behaviors, sensory factors, and psychological readiness [4, 65, 69]. Dietitians, as members of the MDT that also includes gastroenterologists and other allied health staff including psychologists [36], play a critical role in individualizing care plans, monitoring progress, including objective markers of nutrition status, and providing ongoing support throughout this process [69].

#### 2.4.2 Parenteral Nutrition

Parenteral nutrition (PN) is a well‐established life‐saving treatment indicated in intestinal failure [71, 72]. PN bypasses the gastrointestinal tract, allowing the provision of nutrition in patients who have compromised digestive systems [73]. While appropriate use of PN can be lifesaving, its use is burdensome and costly and can cause significant complications. They include risks of infections associated with catheter use, metabolic derangements, and potential liver disease due to long‐term reliance on intravenous nutrient delivery [74]. Therefore, PN is not an appropriate treatment modality for patients who retain normal gastrointestinal function, where oral or enteral feeding should be prioritized to support digestive health and maintain physiological gut function [75].

Indeed, consistent with the above considerations, a position paper from the European Society of Clinical Nutrition and Metabolism, the European Society of Neurogastroenterology and Motility, and the Rome Foundation for DGBI articulated the consensus opinion [4] that long‐term (home) PN should not be prescribed for patients without intestinal failure, where the oral and/or enteral route can be utilized. This approach is also supported by the Small Bowel and Nutrition Committee and the Neurogastroenterology and Motility Committee of the British Society of Gastroenterology [4].

Thus, PN should only be prescribed in exceptional circumstances for a time‐limited period to achieve nutritional safety, while the multi‐disciplinary team focuses on appropriate biopsychosocial holistic and rehabilitative approaches to manage the patient's primary underlying condition.

### 2.5 Pharmacologic and Psychological Interventions

Statement 20 The medical treatment of patients with DGBI‐AFIB should be directed at addressing the main symptom(s), while utilizing the minimum number of medications necessary.

Level of Evidence: V Grade of Recommendation: D Accepted by 92.3%.

Statement 21 Treatment of symptoms with opioids should be avoided in the management of patients with DGBI‐AFIB.

Level of Evidence: V Grade of Recommendation: D Accepted by 92.3%.

If reassurance fails, pharmacotherapy, dietary interventions, psychotherapy, and behavioral interventions, specifically brain‐gut behavior therapies [76] are typically considered for patients with DGBI.

The data presented here represents the best available guidance for managing these patients. In the absence of controlled trials specifically addressing AFIB in DGBI, findings from other DGBI studies can inform treatment, albeit cautiously. For pediatric patients, relevant guidelines on the use of pharmacologic and psychological interventions should be consulted [14, 77].

#### 2.5.1 Prokinetics

In the clinical setting, prokinetics including metoclopramide and domperidone are widely used and have been shown to improve symptoms in FD [78], however, trials are needed to assess their efficacy in DGBI‐AFIB.

#### 2.5.2 Psychotropic Drugs

Specific psychotropic drugs (e.g., neuromodulators) have shown efficacy in alleviating visceral pain and improving quality of life in IBS and FD which often present with meal‐related symptoms [79]. However, in a recent systematic review, only tricyclic antidepressants (TCAs), but not serotonin reuptake inhibitors were found to be effective [80, 81]. TCAs can be effective for DGBI in low doses but benefits often take months to emerge and side effects may lead to early treatment discontinuation [82].

Special consideration can be given to the psychotropic drug mirtazapine, which has good therapeutic effects in patients with FD and weight loss [83]. In a randomized trial, 8‐week treatment with mirtazapine significantly improved the overall severity of dyspeptic symptoms, gastrointestinal‐specific anxiety, quality of life, and increased weight in patients with FD [84].

#### 2.5.3 Spasmolytics

Peppermint oil is considered beneficial for patients with DGBI based on studies in patients with FD [85] and IBS [86]. Another antispasmodic used for patients with IBS and meal‐related pain is Mebeverine [87, 88].

#### 2.5.4 Psychotherapeutic Interventions

Statement 22 Psychosocial and behavioral issues frequently influence the manifestation of symptoms, in DGBI‐AFIB, highlighting the necessity for timely access to specialist psychosocial support.

Level of Evidence: V; Grade of Recommendation: D; Accepted by 92.3%.

Patients with DGBI‐AFIB often experience heightened anxiety or stress, or low mood related to food intake. Therefore, incorporating psychotherapeutic strategies is essential for managing these health‐related comorbidities, as well as addressing underlying belief systems which potentially amplify symptom severity (Statement 22).

Several psychological therapies used in DGBI show equivalent efficacy (RR 0.71–0.79) [89] with Cognitive Behavioral Therapy (CBT) and gut‐directed clinical hypnotherapy having the most substantial evidence for IBS [89, 90]. CBT is frequently part of multidisciplinary treatment approaches for DGBI [36, 58]. CBT for DGBI focuses on improving patient skills to manage health‐related anxiety and stress, for example, in IBS [89, 90]. CBT can also use exposure techniques to gradually reintroduce food stimuli thought to trigger symptoms, under medical supervision [91, 92]. Pilot data indicate this treatment is feasible for patients with DGBI‐AFIB [93].

*Gut‐directed Hypnotherapy* combines traditional psychologically‐based cognitive and muscle relaxation techniques, with personalized hypnotic suggestions aimed at individual gut symptoms, promoting better control or reduction of unpleasant symptoms [94]. Efficacy has been tested mainly in IBS, with variable quality across studies [90]. In children, this modality has shown the greatest efficacy for the management of IBS and functional abdominal pain [14].

Often used in conjunction with CBT, *Mindfulness‐based therapies* have been more recently introduced as a possible psychological treatment for DGBI to promote stress relief and alter the perception of gastrointestinal symptoms. Of the studies conducted in people with IBS, data suggest that Mindfulness‐Based Stress Reduction training was associated with improvements in gastrointestinal symptoms [95, 96, 97]. The specific Acceptance and Commitment Therapy approach that also utilizes components of mindfulness has some early evidence for reducing IBS symptoms and anxiety or depression, but efficacy data are currently limited [98, 99].

Treating DGBI‐AFIB involves a combination of general measures, reassurance, dietary interventions, pharmacotherapy, and psychotherapeutic interventions, but clinical trials for this patient cohort are lacking. Promising options include dietary interventions, prokinetics, neuromodulators, and tailored psychological support. Future research is warranted to establish definitive treatment protocols for this underserved population.

### 2.6 Specific Considerations for Children and Adolescents

Statement 23 A multidisciplinary integrated care model is recommended to manage *pediatric patients* with DGBI‐AFIB effectively. At minimum, this model should include a dietitian, psychologist or psychiatrist, general pediatrician/physician (for patients under 12), and gastroenterologist, with involvement from an adolescent medicine physician for those aged 13 to 18.

Level of Evidence: V; Grade of Recommendation: D; Accepted by 84.6%.

In children with feeding problems, disordered eating behaviors and DGBI are common [100, 101, 102]. Feeding problems are more common in younger children than in older children and adolescents, with functional disorders being more prevalent [101, 103].

In adolescents, differentiation between DGBI‐induced altered food intake behavior and eating disorders can be challenging due to ongoing development, evolving self‐awareness, and parent–child dynamics [104]. Many adolescents with complex DGBI‐AFIB may later be treated for eating disorder symptoms after transitioning to adult care [105].

Moreover, in childhood and adolescence, rumination has been identified as an important mechanism of altered food intake, often overlapping with FD rather than occurring in isolation. In a study of 76 children with rumination linked to persistent fore‐gut symptoms, 35% had weight loss [106]. Programs targeted at managing rumination have helped reduce reliance on artificial nutritional support, while any delays in diagnosis can hinder decreased treatment success [107]. Therefore, early consideration of this entity in children with persistent regurgitation and “vomiting” is important.

In childhood and adolescence, chronic malnutrition can have significant or permanent complications relating to puberty, growth, development, and bone mineralization. While artificial feeding methods remain best avoided, these considerations add complexity to decision‐making around the mode and duration of nutrition support. Most importantly, they further highlight the need for referral to tertiary MDTs and the need for further research. Furthermore, issues of informed consent, patient autonomy, and duty of care are also made more complex by the developmental stage of children and adolescents and the parent–child relationships.

Altering diets to improve or resolve symptoms is extremely common across the spectrum of pediatric DGBI [108]. Some studies show that up to 93% of patients with DGBI identify at least one food and/or food type as worsening gastrointestinal symptoms, without evidence of food allergy [20, 109, 110, 111, 112]. As a result, children with functional abdominal pain disorders (now referred to as pain‐related DGBIs) or their health practitioners frequently implement additional dietary restrictions or exclusions [113, 114].

ARFID is also recognized in children and adolescents, with a Canadian study showing the highest incidence among those aged 10–14 years (3.43/100000) and girls aged 15–18 years (2.24/100000) [115]. A higher prevalence of ARFID has been reported in children with DGBI, especially FD [116], with some studies demonstrating that in 23% of pediatric neurogastroenterology patients avoidant and restrictive food intake behavior [117]. While this altered food intake behavior is likely explained by meal‐related symptoms, there is a risk that these patients with DGBI are misdiagnosed as having an eating disorder including ARFID.

Therefore, before dietary interventions are recommended, a careful assessment of the child's and family's situation and dietary intake needs to be performed [14]. Restrictive diets may be inappropriate if excessive food restriction is already present [117, 118]. Additionally, the route of feeding should be thoughtfully considered, as enteral, or especially parenteral, nutrition in adults has not shown long‐term improvements in symptoms or nutrition but is associated with complications [67]. In pediatric DGBI, such feeding routes should not be routinely considered.

A multidisciplinary integrated care model is recommended for managing children with DGBI‐AFIB, ideally consisting of a dietitian, psychologist or psychiatrist, general pediatrician and/or physician, and gastroenterologist. A general pediatrician should assess patients under 12 years, while an adolescent medicine physician (when available) can be involved for ages 13 to 18 years. Adolescent physicians can address broader psychosocial and mental health factors, including family dynamics, school relationships, and peer interactions, and coordinate care with school wellbeing coordinators, community clinicians, National Disability Insurance Scheme case managers, and General Practitioners (Statement 20). Where available, contemporary guidelines for the management of DGBIs in children should be used to manage symptoms [14, 77].

### 2.7 Managing Expectations of Consumers

Patients affected by severe DGBI‐AFIB often struggle to maintain work, study, and social interactions and suffer high rates of distress and mental illness [37]. At the same time, patient expectations are increasingly shaped by information obtained online. This information is frequently not backed by scientific evidence.

In recent years, many patients display a strong sense of identity and belonging to online peer groups through their illness, termed social media‐associated abnormal illness behavior [119, 120]. In conditions such as DGBI that are not defined by structural abnormalities, and with frequent dismissal of their disorders as being caused by psychiatric conditions, patients face stigma that can lead to emotional distress and difficulties in accessing appropriate care. This can cascade into a search for organic causes that may further complicate care, especially in fragmented healthcare settings where patients may consult multiple healthcare providers over time. The increased healthcare utilization and risk of iatrogenic harm [4] in these circumstances are stressful for patients and present a significant challenge for healthcare providers. In this context, establishing a strong rapport with patients is essential. Healthcare providers should prioritize active listening to fully understand patients' concerns and validate their feelings, while also guiding them toward evidence‐based information with a focus on patient‐centered education and communication. Involving patients in the decision‐making process surrounding their care is vital. This collaborative approach fosters trust and ensures that patients are aware of available clinical guidelines. Additionally, seeking a second independent opinion from another healthcare professional can be beneficial. A key role is played by the primary care physician, who needs to be well‐informed regarding the management plan to ensure appropriate support in the primary care setting.

### 2.8 Knowledge Gaps and Future Research Directions

Recognizing DGBI‐AFIB as a newly defined condition (and its intersection with eating disorders) also highlights existing knowledge gaps. Research thus should investigate the mechanisms linking gastrointestinal symptoms with systemic comorbidities, while clinical studies should focus on diagnostic modalities and interventions that address gastrointestinal and extraintestinal problems (Table 3). Additionally, exploring biomarkers and genetic factors can help develop personalized treatment approaches. Furthermore, the recognition of DGBI‐AFIB as a distinct entity emphasizes the need for collaboration among gastroenterologists and various other disciplines to improve patient outcomes. There is a pressing need for DGBI‐AFID specific studies to be able to provide high quality treatments in the routine clinical setting.

**TABLE 3:** Knowledge gaps and future research directions.

Clinical assessment	Validation of existing diagnostic criteria and development of standardized assessments to appropriately guide therapy, characterize symptoms and impact on patients' quality of life with disorders of gut‐brain interaction and altered food intake behavior (AFIB). The current DGBI‐AFIB criterion of ≥ 5% weight loss over 6–12 months is not specific to DGBI‐AFIB; future prospective studies should refine it by incorporating symptom trajectories and objective eating‐behavior measures.
Risk factors for the progression	Patients presenting with DGBI‐AFIB likely present only a fraction of patients with meal‐related symptoms. Thus, risk factors for the progression of DGBI complicated by severely altered food intake and nutritional deficiencies need to be identified.
Development and validation of screening tools and cut‐offs	The development and validation of screening tools specific to this DGBI population are critically needed. These tools should include clinically relevant cut‐offs to improve the accuracy of assessments and facilitate early detection of disorders. Effective screening instruments will ensure timely intervention, which is crucial for individuals experiencing complex interactions between gastrointestinal and psychological symptoms.
Define the role of comorbidities in disease progression	It is essential to better understand the role of comorbidities, including psychiatric comorbidities, in manifesting, progressing, and responding to therapeutic interventions. Research should aim to identify specific comorbid conditions, their prevalence, and how these relationships change over time. Recognizing predictors of these comorbidities can inform targeted interventions and management strategies tailored to each patient's psychological needs.
Adapt and validate therapeutic interventions (medical, dietary, psychological)	Utilizing appropriately powered and designed clinical studies to address the unique needs of these patients
Define long‐term outcomes of patients who utilized oral feeding, enteral or parenteral feeding	Studying clinical cohorts to inform the future development of prospective randomized clinical trials.
Defining patient needs and expectations	Conduct qualitative studies on the lived experiences of patients with DGBI‐AFIB, including interviews and focus groups, to inform evidence‐based statements.

## 3 Conclusions

In recent years, the number of adults and children with severe meal‐related symptoms and subsequently altered (reduced) food intake appears to be increasing. These patients frequently meet established criteria for DGBI (and in particular FD) with severe meal‐related symptoms triggering alterations of food intake that ultimately result in weight loss and nutritional deficiencies.

Individuals with DGBI‐AFIB also frequently suffer from psychological comorbidities such as depression and anxiety, and there may also be increased association with rare systemic disorders HSD, POTS, and MCAS in this subset, diagnoses of which are often made on clinical suspicion or self‐diagnosis. Diagnosis of these comorbid conditions in DGBI‐AFIB remains challenging and should be validated by appropriately designed prospective clinical studies utilizing established diagnostic criteria for these co‐morbidities.

Patients experiencing severe meal‐related symptoms that are not explained by structural abnormalities face stigma that can cause emotional distress and difficulties in accessing appropriate care. This can trigger a vicious circle that may exacerbate symptoms and cascade into a search for organic causes, which may further complicate care, especially in fragmented healthcare settings where patients consult multiple specialists over time. A lack of coordinated care increases the risk that well‐meaning medical or dietary interventions may cause harm and further exacerbate the situation. Effective management begins by making a positive diagnosis of a DGBI‐AFIB by ruling out organic causes, adhering to Rome Criteria and evaluating for eating disorders where appropriate.

Long‐term management requires an iMDT approach which provides empathetic care, education, realistic expectations, and a collaborative treatment plan. Treatment options, both pharmacological and non‐pharmacological, should be tailored to the predominant symptoms and prior treatment responses. Pharmacotherapy may target the gastrointestinal tract, enteric nervous system, or central nervous system, while non‐pharmacological strategies should adhere to established DGBI principles, potentially incorporating CBT or similar approaches.

Oral nutrition support should be prioritized and personalized by an experienced dietitian. Invasive treatments like gastric or post‐pyloric tube feeding or PN should be avoided unless necessary to address short‐term, life‐threatening, severe nutritional deficiencies. If used, these treatments should be as brief as possible and conducted in an MDT setting.

Patients with DGBI‐AFIB frequently “slip through diagnostic and therapeutic gaps”. DGBI‐AFIB patients have the same complex multi‐disciplinary needs as patients with eating disorders; however, they do not meet full criteria for formal eating disorders or ARFID. As a result, such patients are not appropriate for eating disorder treatment programs which target eating disorder behaviors and cognitions such as body dysmorphia. Patients with DGBI‐AFIB, in contrast to eating disorder populations, do not generally have body dysmorphia and, in contrast to the eating disorder population, desperately want to gain weight; however, they feel powerless to do so due to the presence of aversive GI symptoms associated with eating.

As such, altered food intake driven by meal‐related symptoms in patients with DGBI constitutes a clinically meaningful condition that warrants its own diagnosis. Formal recognition would improve diagnostic clarity, guide multidisciplinary management (including nutrition, psychology, and gastroenterology), enable standardized assessment and outcome measurement, and underpin targeted interventions. While clinical trials exploring the safety and efficacy of specific treatments, especially pharmacologic and psychotherapeutic interventions, for patients with DGBI‐AFIB are scarce, it is reasonable to extrapolate from available evidence for patients with DGBI until more specific evidence is available. All treatments should be coordinated with primary care to ensure optimal long‐term management. Given the significant burden of these conditions on patients, further research is essential to better characterize the disease mechanisms and develop effective therapeutic interventions for those affected with DGBI‐AFIB.

Finally, we acknowledge that the recommendations provided in this Clinical Practice Update, including the proposed definition and criteria for DGBI‐AFIB, are derived from an expert‐informed Delphi consensus process. It is hoped that this update will serve as a foundation to guide future research and clinical practice, supporting the further development and refinement of a condition that is highly problematic for patients and clinicians yet currently lacks a strong evidence base and clear clinical framework.

## Author Contributions

Study design/conception/writing the first draft of the manuscript: A.S. and G.H. All authors contributed to writing the final draft and approved the final version.

## Funding

National Health and Medical Research Council grants (Ideas APP1084544, Centre for Research Excellence in Transforming Gut Health APP170993).

## Conflicts of Interest

Nicholas Talley: Brown University, Agency for Health Care Research and Quality (fiber and laxation) (2024), Rome Foundation (member gastroduodenal committee) (present), Biocodex (FD diagnostic tool) (2024), Microba (consulting microbiome 2025), Comvita Manuka Honey (FD trial consulting) (2025), BluMaiden (microbiome), and Intrinsic Medicine (research support) outside the submitted work. In addition, Dr. Talley has a patent Nepean Dyspepsia Index (NDI) 1998, a patent Licensing Questionnaires Talley Bowel Disease Questionnaire licensed to Mayo/Talley, “Diagnostic marker for functional gastrointestinal disorders” Australian Provisional Patent Application 2,021,901,692, “Methods” and compositions for treating age‐related neurodegenerative disease associated with dysbiosis US Patent Application No. 63/537,725, “Compositions and methods for the treatment of oesophageal disorders” Australian Provisional Patent ID 2025902002. Gemma Sharp: Founding Director of the charity, Consortium for Research in Eating Disorders (CoRe‐ED). The other authors declare no conflicts of interest.

## Data Availability

Data sharing not applicable to this article as no datasets were generated or analysed during the current study.
